# The Pesticide Exposure of People Living in Agricultural Community, Northern Thailand

**DOI:** 10.1155/2018/4168034

**Published:** 2018-11-29

**Authors:** Anurak Wongta, Nootchakarn Sawarng, Phannika Tongchai, Kunrunya Sutan, Tanyaporn Kerdnoi, Tippawan Prapamontol, Surat Hongsibsong

**Affiliations:** ^1^NCD Center of Excellence, Environment and Health Research Unit, Research Institute for Health Sciences, Chiang Mai University, Chiang Mai 50200, Thailand; ^2^Environmental Science Ph.D. Program, Faculty of Science, Chiang Mai University, Chiang Mai 50200, Thailand; ^3^Faculty of Public Health, Chiang Mai University, Chiang Mai 50200, Thailand

## Abstract

**Background and Aim:**

Biomarkers of pesticide exposure are generally lacking in communities where agricultural crops are grown. The purpose of this study was to focus on evaluating biomarkers of pesticide exposure in people living in an agricultural area of San Pa Tong District in Chiang Mai Province, northern Thailand.

**Materials and Methods:**

One hundred and twenty-four participants (38 nonfarm workers, 38 rice growers, 31 longan growers, and 17 vegetable growers) from San Pa Tong District gave consent to participate in the study. Pesticide exposure was assessed by determining acetylcholinesterase (AChE) and butyrylcholinesterase (BChE) levels in blood samples using Ellman's method and measuring 6-dialkylphosphate metabolites (DAPs), 3-phenoxybenzoic acid (3-PBA), and glyphosate in urine samples using chromatographic methods.

**Results:**

AChE and BChE activities in the nonfarm worker group had higher level than those in the grower groups. DAPs were detected in almost all urine samples and 3-PBA was detected in 12-45% of each group, while glyphosate was found in 11 – 30% among the three groups of growers but not in nonfarm workers.

**Conclusion:**

In this study, participants living in an agricultural area of San Pa Tong District were exposed to organophosphate, synthetic pyrethroid, and glyphosate through multiple pathways.

## 1. Introduction

Agriculture remains a large and important sector in the Thai economy, including a high proportion of the current labour force working in this sector. Current agricultural practices see pesticides being widely used for protecting crops and increasing yields. The most commonly used insecticides in Thailand are organophosphates and synthetic pyrethroids [1, 2], and the most used herbicide is glyphosate [3]. San Pa Tong District is a suburban district in Chiang Mai Province, northern Thailand, producing several kinds of agricultural crops such as rice, longan, and onions. Pesticide use is common in this district.

Biomarkers of pesticide exposure used to assess pesticide exposure include: (1) cholinesterase enzyme activities including acetylcholinesterase (AChE) and butyrylcholinesterase (BChE) for assessing the exposure to organophosphate and carbamate containing pesticides; (2) dialkyl phosphate metabolites (DAPs), nonspecific for organophosphates [4–7]; (3) urinary 3-phenoxybenzoic acid (3-PBA), a major metabolite of synthetic pyrethroid[8, 9]; and (4) urinary glyphosate [10].

Most recent studies in Thailand have reported exposure to a single type of pesticide in single crops grown by farmers, such as urinary DAPs levels in rice [11] and chili [12] or general farmers [13]. Despite the fact that Thai farmers use multiple pesticides in multicrop cultivation, the biomarker of pesticide exposure is lacking in one community of agricultural area where several agricultural crops are grown.

The hypothesis of this study was the community where multiple pesticides in multicrop cultivations were used; the people will be exposed to more than one kind of pesticide. So, the purpose of this study was to evaluate the biomarkers of pesticide exposure in people living in an agricultural area of San Pa Tong District, Chiang Mai Province, northern Thailand. Cholinesterase activity, levels of dialkyl phosphate (DAPs) metabolites, 3-phenoxybenzoic acid (3-PBA), and glyphosate in urine were analyzed and used as biomarkers of exposure to organophosphate, pyrethroid, and glyphosate pesticides, respectively. The results from this study will support a training program which aimed at increasing health awareness among farm and nonfarm workers.

## 2. Materials and Methods

### 2.1. Chemicals and Reagents

All organic solvents (acetone, ethyl acetate, acetonitrile, hexane, and toluene) were of analytical grade and were purchased from J.T. Baker (PA, USA). Acetylthiocholine iodide and butyrylthiocholine iodide (Sigma–Aldrich (MO, USA)) and Pentafluorobenzyl bromide (PFBBr, 99%, Sigma–Aldrich (MO, USA)) were used. The following chemical standards were used: dimethyl phosphate (DMP, 98%, ACROS Organic), diethyl phosphate (DEP, 99.5%, Chem Service), dimethyl thiophosphate (DMTP, >90%, Cerilliant), dimethyl dithiophosphate (DMDTP, >90%, Cerilliant), diethyl dithiophosphate (DEDTP, 90%, Sigma), diethyl thiophosphate (DETP, 98%, Sigma), 3-PBA (98%, Fluka), and glyphosate (99%, Chem Service). The derivatized 1,1,1,3,3,3-Hexafluoroisopropanol (HFIP), N, N'-Di-isopropyl carbodiimide (DIC), and 9-fluorenylmethyloxycarbonyl chloride (FMOC-Cl) were from Sigma-Aldrich (MO, USA).

### 2.2. Urine and Blood Samples Collection

First morning urine samples were collected in 250 ml urine containers and stored in an ice cooler from collection through transportation to the laboratory. The samples were aliquoted to 15 ml ×3 tubes and frozen at -20°C until they were analyzed. Venipuncture was used to collect blood samples (3 ml) from participants by trained staff, into sodium-heparinized tubes. The blood was spun and separated to collect plasma. The red cells were washed by cooled phosphate buffer saline, pH 7.4. The samples were aliquoted and frozen at -20°C prior to measuring cholinesterase enzyme activity.

### 2.3. Study Population

Inclusion criteria targeted four groups, nonfarm workers, rice growers, longan growers, and vegetable growers, aged between 18 and 65, who had lived for at least one year in San Pa Tong District, Chiang Mai Province, Thailand. Demographic data were collected through a standardized survey questionnaire covering age, education status, personal income, etc. Questionnaire data and biological samples were collected during August and October 2017.

This study was approved by the Human Ethical Review Committee, Research Institute for Health Sciences, Chiang Mai University, Thailand (Certificate no. 3/2561), on 06 January 2017. All participants provided written informed consent to be interviewed and agreed to the collection of blood and urine samples.

### 2.4. Determination of Biomarkers of Pesticide Exposure

#### 2.4.1. Measurement of AChE Activity

AChE and the BChE activities were analyzed using a modified version of Ellman et al. methods [14]. The cholinesterase activity was measured by using acetylthiocholine iodide and butyrylthiocholine iodide as substrates for measuring the AChE activity in red blood cells and the BChE activity in plasma, respectively, and reported as units per milliliter (U/mL).

#### 2.4.2. Determination of DAPs in Urine Samples

DAP is a nonspecific metabolite of OPs which was widely used for assessing exposure to OPs from several routes, that is, oral, dermal, and inhalation. Once OP pesticide exposure occurs, it is metabolized via dealkylation, hydrolysis, and isomerization. DAPs in the urine can be used indirectly measuring OP pesticide exposure. Urinary DAPs were measured using the method of Prapamontol et al [15]. The DAPs were analyzed using a Hewlett-Packard 7890B-flame photometric detector (GC-FPD) and 7693 Autosampler (Agilent Technology, CA, USA) (Agilent Technology, CA, USA) equipped with HP-5 (30 m × 0.25 mm.id, 0.25 um film thickness) columns. The analysis included six nonspecific metabolites of organophosphates pesticides, that is, DAPs derivatives consisting of DMP, DEP, DMTP, DMDTP, DEDTP, and DETP. Sum variables were calculated: sumDEP: DEP + DETP+DEDTP, sumDMP: DMP+DMTP + DMDTP, sumDAP: sumDEP + sumDMP. The DAPs concentrations were adjusted for creatinine concentrations and converted from *μ*g/L to *μ*g/g creatinine. The creatinine in urine was determined by Jaffe's reaction.

#### 2.4.3. Determination of 3-PBA in Urine Samples

The method for detecting 3-PBA in urine samples was modified from Pakvilai et al.[16] The 3-PBA was analyzed by using Hewlett-Packard 7890B-electron capture detector (GC-ECD) and Autosampler G4513A (Agilent Technology, CA, USA) equipped with HP-5 (5% phenylmethyl polysiloxane with 30 m × 0.25 mm, 0.25 *µ*m film thickness) after derivatization by HFIP and DIC.

#### 2.4.4. Determination of Glyphosate in Urine Samples

Glyphosate in the urine samples was measured using a previously published method [10]. In brief, urine samples were derivatized by FMOC-Cl prior to analysis using high performance liquid chromatography (HPLC: Agilent, 1100) equipped with fluorescence detector (FLD: Agilent, 1046A). The fluorescence was set at excitation 242 nm and emission 388 nm.

### 2.5. Quality Control

Pooled blood samples were prepared from the blood of all participants. Red blood cells and plasma samples were separated, aliquoted, and stored at -20°C prior to analyzing the samples as quality controls. The pooled red blood cells and plasma samples were used for measuring the accuracy and precision of the methods and reported as coefficient variation, inter- and intrabatch coefficients.

Pooled urine samples were also created from a random selection of participant samples. After analyzing background levels, the pooled urine was spiked with various levels of DAPs, 3-PBA, and glyphosate to calculate the accuracy, precision, and recovery of each biomarker for ensuring accurate quantification of real samples. The spiked urine samples were subjected to the same extraction and analysis procedures as real samples and calculated for inter- and intrabatch coefficients.

### 2.6. Quantification of Methods for Detecting Biomarkers of Pesticide Exposure

The methods for determining AChE, BChE, 6 DAPs, 3-PBA, and glyphosate are shown in Table 1. The methods had acceptable values of parameters for analyzing in all blood and urine samples.

### 2.7. Statistical Analysis

The collected data were analyzed using SPSS. Data on demographic characteristics were reported as percentages. The level of biomarker of exposure was reported in mean, standard deviation, and geometric mean. The nonnormal distribution data were transformed to log normal before analyzing independent samples with ANOVA to compare the level between groups of subjects at the significance level of 0.05.

## 3. Results and Discussion

### 3.1. Demographic Data

The demographic characteristics of the 124 participants are presented in Table 2. The participants were nonfarm workers, rice growers, longan growers, and vegetable growers. Most of the growers were male and married. Most of the participants were older than 40 years, had primary school as their highest level of education, and had personal incomes lower than 10,000 Thai Baht (lower than $300) per month.

### 3.2. Cholinesterase Activity as Biomarkers of OPs and Carbamate Pesticide Exposure in Blood

The results of AChE and BChE activity are shown in Table 3. AChE and BChE in blood samples showed no significant difference between nonfarm workers and rice growers. However, nonfarm workers and rice growers had significantly higher (p<0.05) AChE activity than longan growers and vegetable growers. BChE in nonfarm workers and longan growers were significantly higher than those in vegetable growers. The results of AChE, BChE, 6 DAPs, 3-PBA, and glyphosate were compared with the results reported in previous studies among a similar population. Longan and vegetable growers had lower AChE activity compared with nonfarm workers and rice growers at p<0.05. There were no significant differences in AChE and BChE between nonfarm workers and rice growers' group, which may suggest equal exposure to OPs and carbamate insecticides. It is apparent that exposure to OPs or carbamates leads to poisoning via the overstimulations of signal transduction due to accumulation of ACh at the synapse [17]. The AChE and BChE activities in every group were in a similar range which was reported in Malaysian by Chan et al. [18] This study also reported higher activities of both AChE and BChE than Hongsibsong et al. who reported among vegetable growers in Thailand [19]. In this study, BChE activity in the three growers' groups was lower than that in nonfarm workers. This result was similar to the study of Pongraveevongsa and Ruangyuttikarn [20].

### 3.3. Dialkyl phosphate Metabolites as Biomarkers of OPs Insecticides Exposure in Urine

The level of DAPs is presented in Table 4. More than 90% of all groups of growers and nonfarm workers had at least one DAP metabolite present in their urine. DEP was found more often in the urine of nonfarm workers than in rice, longan, and vegetable growers but at a lower concentration. DMDTP was not detected among longan growers. The level of DMP was the highest among nonfarm workers and rice and longan growers while the level of DMTP was the highest in vegetable growers. This shows that nonfarm workers were exposed to similar kinds of OPs as rice and longan growers. There was a low level of 6 DAPs in nonfarm workers. Organophosphate pesticide metabolites with ethyl moieties (DEP, DETP, and DEDTP) were detected in the rice and vegetable growers at lower concentrations than dimethyl moieties (DMP, DMTP, and DMDTP), but not in nonfarm-workers or longan growers. Total dialkyl phosphates (∑DAP) levels in the longan growers' group were the highest followed by rice growers, vegetable growers, and nonfarm workers, respectively.

The level of DAPs in nonfarm workers was higher than that in the other groups but the percent of detection was lower; this may indicate recent expose to methyl OPs from consuming fruit or vegetables or being in areas where those pesticides were being used. The most frequently detected DAPs in the present study were DEP followed by DETP, DEDTP, DMTP, DMDTP, and DMP, respectively. This result is different from previous studies which reported that the most commonly detected metabolite was DMP. This may be due to different kinds of OPs used in each area [21, 22].

DAPs concentrations were 2 times higher in rice growers in the present study compared to rice growers in another province of Thailand [23] and to school-aged children in a rice-growing area in northeastern Thailand [11]. The higher concentrations found in the present study compared to previous studies may be due to the differences in the areas, age of subjects, type of target insects, type of pesticides used for crop protection, and the behavior of growers in each area. However, the present study showed lower levels of DAPs than those reported in a study of a Chinese population of Shanghai (Eastern China). [24]

### 3.4. 3-Phenoxybenzoic Acid as Biomarkers of Pyrethroid Insecticides Exposure in Urine

Table 5 showed the level of urinary 3-PBA and glyphosate among subjects who worked in different cropping systems. The detection of 3-PBA in urine samples of nonfarm workers and rice, longan, and vegetable growers was 36.8%, 44.7%, 30.0%, and 11.8%, respectively. The highest GM concentration of 3-PBA was found in longan growers (27.52 ng/ml) and the lowest was found in vegetable growers (3.29 ng/ml). Vegetable growers had significantly lower (p<0.05) 3-PBA concentrations than in nonfarm workers and rice and longan growers. The percent of detection of 3-PBA in the urine was lower than that in a study by Thiphom et al.[25], who studied concentrations in consumers and farmers in Chiang Mai, Thailand, possibly due to different crops and different areas. Rice and longan growers appeared to have higher exposure, most likely due to their respective agricultural activities [26]. Nonfarm workers who live in agricultural communities also likely had higher exposure to insecticides. [27] Since the group of vegetable growers had the lowest level of 3-PBA, the exposure may be from various pathways such as consumption of pesticide-contaminated vegetables and fruits, dermal contact, and inhalation.

### 3.5. Glyphosate as Biomarkers of Glyphosate Herbicide Exposure in Urine

Glyphosate was detected at low levels; urinary glyphosate was detected in 30% of longan growers (with the highest amount among all groups: 2.92 ng/ml), 10% in rice growers, and 23.5% in vegetable growers. However, glyphosate was not detected in the urine of nonfarm workers. The geometric means of glyphosate concentrations in the three grower groups were lower than those found in a cohort of general farmers from a previous study in Thailand by Hongsibsong et al. [10] but higher than the mean level found in German adults from 2001 to 2015 [28] and the maximum value found in Irish horticultural workers [29]. This may be from patterns or types of herbicides used in those areas.

## 4. Conclusions

People who work on their farms and live in agricultural areas of San Pa Tong District, Chiang Mai, Thailand, are exposed to organophosphate, synthetic pyrethroid, and glyphosate pesticides. The present study also demonstrated multipesticide exposure in different groups of people living in this farming community. This is a new and significant finding. The level of organophosphate, synthetic pyrethroid, and glyphosate exposure in growers was higher than in nonfarm workers. The exposure to the herbicide glyphosate was not found in nonfarm workers but was found in the grower's groups. Growers having different levels of pesticide exposure from high to low exposure were longan, rice, and vegetable growers, respectively. This points to the need for self-protection from exposure to pesticides by growers, and for growers in particular to consider preventive options such as pesticide substitution using certain compounds, reducing use, or switching to organic farming. The data from the present study adds new information about exposure to multiple elements and can be used for further studies in similar farming communities to examine the impact of patterns of exposure.

## Figures and Tables

**Table 1:** Quantification of methods for detecting biomarkers of pesticide exposure.

Metabolites	LOD (*µ*g/L)	Recovery (%)	Interbatch (%RSD)	Intrabatch (%RSD)
AChE activity	-	-	6.2	12.3
BChE activity	-	-	3.1	9.11
6 DAPs	50-125	76.0 – 112	6.2-14.6	7.1-22.5
3-PBA	1.0	93.0	3.2	3.8
Glyphosate	0.5	80.0	3.0	8.9

AChE: acetylcholinesterase; BChE: butyrylcholinesterase; 6 DAPs are dimethyl phosphate, dimethyl thiophosphate, dimethyl dithiophosphate, diethyl phosphate, diethyl thiophosphate, and diethyl dithiophosphate; 3-PBA: 3-phenoxybenzoic acid.

**Table 2:** Percentage distribution of sociodemographic characteristics, by study group status.

	Nonfarm worker (n=38) %	Grower groups		
		Rice (n=38) %	Longan (n=31) %	Vegetable (n=17) %
Gender				
* Male*	42	74	94	82
* Female*	58	26	6	18

Status				
* Single*	13	0	3	12
* Married*	84	92	90	88
* Widowed*	3	8	6	0

Age (years)				
* 18-25*	8	5	0	0
* 26-40*	29	8	19	6
* 41-60*	50	47	42	59
* 61-65*	13	39	35	35

Education				
* Primary*	42	61	61	59
* Secondary*	11	11	6	18
* High school*	26	18	16	18
* Bachelor*	21	5	13	6
* No formal education*	0	5	0	0

Income (Baht)				
* Less than 10,000*	66	79	71	88
* 10,001-20,000*	24	18	13	6
* 20,001+*	11	3	13	6

**Table 3:** Cholinesterase activity (mean and standard deviation (SD)) of nonfarm workers and grower groups.

Cholinesterase enzyme	Nonfarm worker	Grower groups		
		Rice	Longan	Vegetable
	Mean ±SD	Mean ±SD	Mean ±SD	Mean ±SD
Acetylcholinesterase	5.11 ± 1.74 ^a,c^	5.29 ± 1.43 ^b,d^	4.28 ± 1.72 ^a,b^	4.35 ± 1.08 ^c,d^
Butyrylcholinesterase	3.28 ± 0.66 ^a^	3.01 ± 0.76	3.25± 0.96 ^b^	2.64 ± 0.72 ^a,b^

*∗*Values followed by the same letters (a, b, c, and d) in the same row are significantly different at p<0.05 by ANOVA after a log-normal transformation.

**Table 4:** Presence of urinary biomarkers of organophosphate pesticides exposure, by study group.

Groups	Urinary biomarkers	Detection (%)	Mean ±SD	Geometric mean
Nonfarm workers	DMP	5.3	19.5±22.1	11.67
	DMTP	36.8	3.39±3.99 ^a^	1.8
	DMDTP	10.5	0.59±0.40	0.48
	DEP	97.4	2.23±1.89 ^c,d^	1.41
	DETP	92.1	1.89±2.51	1.08
	DEDTP	34.2	1.02±1.73 ^g^	0.63
	∑DMs	39.5	5.92±8.92 ^h,i^	2.76
	∑DEs	100	4.26±4.05	3.04
	∑DAPs	100	6.61±7.56	4.19

Rice grower	DMP	15.8	16.1±9.82	12.3
	DMTP	15.8	9.16±14.7	2.87
	DMDTP	7.9	3.24±5.32	0.64
	DEP	94.7	3.84±4.14 ^c^	2.53
	DETP	97.4	1.82±1.99 ^e^	1.23
	DEDTP	31.6	0.71±0.64	0.53
	∑DMs	28.9	14.7±14.4 ^h^	8.2
	∑DEs	100	5.64±5.29	3.91
	∑DAPs	100	9.89±12.1	5.51

Longan grower	DMP	13.3	9.68±5.22	8.65
	DMTP	16.7	1.44±0.72, ^b^	1.32
	DMDTP	0.0		
	DEP	93.3	7.74±11.5, ^d^	3.38
	DETP	83.3	2.46±3.35, ^f^	1.5
	DEDTP	23.3	1.53±3.19	0.5
	∑DMs	26.7	5.74±6.43, ^j^	3.16
	∑DEs	96.7	9.96±13.4, ^k^	4.93
	∑DAP	96.7	11.5±13.3, ^s^	6.14

Vegetable grower	DMP	11.8	9.38±4.78	8.74
	DMTP	11.8	24.8±2.68, ^a,b^	24.7
	DMDTP	5.9	0.61±	0.61
	DEP	88.2	2.18±1.16	1.91
	DETP	76.5	0.82±0.67, ^e,f^	0.62
	DEDTP	11.8	0.22±0.10, ^g^	0.2
	∑DMs	23.5	17.2±9.55, ^I,j^	14.8
	∑DEs	94.1	2.73±1.72, ^k^	2.24
	∑DAPs	94.1	7.03±8.99	4.91

*∗* Values followed by the same letters (a-k) in the same column are significantly different at p<0.05 by ANOVA after a log-normal transformation, GM: geometric mean, LOD: limit of detection, DMP: dimethyl phosphate, DMTP: dimethyl thiophosphate, DMDTP: dimethyl dithiophosphate, DEP: diethyl phosphate, DETP: diethyl thiophosphate, DEDTP: diethyl dithiophosphate, and 3-PBA: 3-phenoxybenzoic acid.

**Table 5:** Urinary biomarkers of synthetic pyrethroid (3-phenoxybenzoic acid: 3-PBA) and glyphosate exposure, by study group.

Groups of subjects			3-PBA	Glyphosate
Nonfarm worker		Detection (%)	36.8	-
		Mean ±SD (ng/ml)	20.3±12.9 ^a^	<LOD
		GM (ng/ml)	16.7	<LOD

Grower groups	Rice	Detection (%)	44.7	10.5
		Mean ±SD (ng/ml)	40.9±45.6 ^b,c^	2.01±0.81
		GM (ng/ml)	27.52	1.89
	Longan	Detection (%)	30	30
		Mean ±SD (ng/ml)	43.8±42.4 ^d^	2.88±1.46
		GM, (ng/ml)	29.8	2.55
	Vegetable	Detection (%)	11.8	23.5
		Mean ±SD (ng/ml)	4.03±3.28 ^a,b,c,d^	3.11±1.15
		GM, (ng/ml)	3.29	2.92

*∗* Values followed by the same letters (a, b, c, and d) in the same column are significantly different at p<0.05 by ANOVA after a log-normal transformation, GM: geometric mean, LOD: limit of detection.

## Data Availability

The demographical data, qualification data, and analyzed concentration of 6 dialkyl phosphate metabolites, 3-phenoxybenzoic acid, and glyphosate data used to support the findings of this study are included within the article.
